# Quality Saving Mechanisms of Mitochondria during Aging in a Fully Time-Dependent Computational Biophysical Model

**DOI:** 10.1371/journal.pone.0146973

**Published:** 2016-01-15

**Authors:** Daniel Mellem, Frank Fischer, Sören Jaspers, Horst Wenck, Michael Rübhausen

**Affiliations:** 1 Center for Free-Electron Laser Science (CFEL), University of Hamburg, Hamburg, Germany; 2 Beiersdorf AG, Applied Biophysics, Hamburg, Germany; University of Pecs Medical School, HUNGARY

## Abstract

Mitochondria are essential for the energy production of eukaryotic cells. During aging mitochondria run through various processes which change their quality in terms of activity, health and metabolic supply. In recent years, many of these processes such as fission and fusion of mitochondria, mitophagy, mitochondrial biogenesis and energy consumption have been subject of research. Based on numerous experimental insights, it was possible to qualify mitochondrial behaviour in computational simulations. Here, we present a new biophysical model based on the approach of Figge et al. in 2012. We introduce exponential decay and growth laws for each mitochondrial process to derive its time-dependent probability during the aging of cells. All mitochondrial processes of the original model are mathematically and biophysically redefined and additional processes are implemented: Mitochondrial fission and fusion is separated into a metabolic outer-membrane part and a protein-related inner-membrane part, a quality-dependent threshold for mitophagy and mitochondrial biogenesis is introduced and processes for activity-dependent internal oxidative stress as well as mitochondrial repair mechanisms are newly included. Our findings reveal a decrease of mitochondrial quality and a fragmentation of the mitochondrial network during aging. Additionally, the model discloses a quality increasing mechanism due to the interplay of the mitophagy and biogenesis cycle and the fission and fusion cycle of mitochondria. It is revealed that decreased mitochondrial repair can be a quality saving process in aged cells. Furthermore, the model finds strategies to sustain the quality of the mitochondrial network in cells with high production rates of reactive oxygen species due to large energy demands. Hence, the model adds new insights to biophysical mechanisms of mitochondrial aging and provides novel understandings of the interdependency of mitochondrial processes.

## Introduction

A detailed comprehension of the functioning of mitochondria is of great concern in miscellaneous scientific disciplines and plays an important role in aging. [2], [3]

The main task of mitochondria is to provide a cell with energy in form of adenosintriphosphate (ATP). In the cytoplasm of the cell glycolysis decomposes glucose in order to gain pyruvate molecules. These molecules are transported into mitochondria where they enter the Krebs cycle [4]. There, the pyruvate reduces NAD+ molecules to the coenzyme NADH. The NADH molecules arrive at the respiration chain where their energy is released gradually by carrying its reducing electrons to lower potentials. The resulting free energy is employed to establish a proton motive force along the inner membrane of the mitochondrium in order to finally transform adenosindisphosphate (ADP) to ATP.

The efficiency of these processes strongly depends on the quality of mitochondria. Their activity can be tracked by the polarization of their mitochondrial membrane potential (MMP). [5] In order to sustain a high membrane potential, Krebs cycle and respiration chain have to be supplied with a sufficient amount of metabolites such as NAD+, ADP or pyruvate. Additionally, the number of defects within the mitochondrial DNA (mtDNA) should be kept at a low level so that newly generated protein complexes involved in the respiration chain or enzymes involved in the Krebs cycle are of high quality. [6, 7]

During the aging of cells mitochondria suffer from self-generated internal reactive oxygen species (ROS) and external oxidative stress produced by other organelles, eventually leading to the death of cells.[8] In order to retain their reliability in energy production mitochondria behave as a very dynamic network that utilizes numerous processes to remain in a state of high quality. [9] Fission and fusion help mitochondria to compensate for rare metabolites, defect mtDNA sequences or damaged protein complexes. [10], [11] Irreversibly damaged mitochondria are excluded from the network by fission processes and afterwards removed and regenerated by a permanent cycle of mitochondrial autophagy (mitophagy) [12] and mitochondrial biogenesis. [13] Repair mechanisms similar to repair procedures of nuclear DNA defects help to maintain the integrity of the mtDNA. [14]

In recent years various experiments provided a better understanding of the processes within the mitochondrial network. [15] Based on this knowledge several *in silico* models have been developed which are considered to generally simulate mitochondrial dynamics. [16, 17] An established model to describe the mitochondrial development during the aging of cells was introduced by Figge et al. in 2012. [1] In this contribution, the probabilistic change of discrete mitochondrial quality states was described by one differential equation which integrated several dynamic mitochondrial processes. Biophysical simulations revealed a decreasing mitochondrial quality and an increasing fragmentation of the mitochondrial network with time. These findings are in good agreement with experimental results *in vitro*. [18, 19] Furthermore, the simulations predicted a deceleration of fission and fusion cycles as a quality saving mechanism in the presence of infectious molecular damage among mitochondria.

Here, we present a new mitochondrial quality model based on the approach of Figge et al. in 2012. We introduce a universal biophysical decay and growth law that defines a time-dependent probability for every mitochondrial process. Furthermore, we adjust and append the mathematical definitions of all mitochondrial processes in the existing model to explicitly adapt them to latest findings in experimental research on mitochondrial networking, recycling, repair and energy consumption [20], [21], [22], [23]. We present and discuss simulations of the mitochondrial quality model.

## Models

Mitochondrial quality depends on the supply with metabolites, the energetic activity and the health of mtDNA and mitochondrial protein complexes. In this model these variables are represented by a single parameter *q* that defines the state of quality of a mitochondrium. The quality *q* is a discrete integer of a value between 0 and *Q*, with *Q* being the maximal quality. A mitochondrium exhibits each quality state with a time-dependent probability *P*(*q*, *t*). For every point in time the sum of the probabilities of all quality states is normalized to 1:

$$\begin{array}{c} N_{\text{prob}}=\sum_{q=0}^{Q}P\left(q,t\right)=1 \end{array}$$ (1)

This system can be described as a homogeneous Markovian model where mitochondrial processes *i* represent the transitions between discrete quality states *q* of a Markov chain. Then, the time-dependence of the probability *P* of quality states can be derived by a Master equation [24]:

$$\begin{array}{c} \frac{dP}{dt}=R\left(t\right)P, \end{array}$$ (2)

where **R**(*t*) is the time-dependent transition matrix with its elements representing all possible transitions between quality states by mitochondrial processes. Due to the conservation norm Eq (1) the probability *P* in total is not altered. Solving the one-dimensional Master equation the change of the probability of a single state q can be described with:

$$\begin{array}{c} \frac{\partial }{\partial t}P\left(q,t\right)=\sum_{q\neq q^{\prime }}\left(R_{q^{\prime }q}P\left(q^{\prime },t\right)-R_{qq^{\prime }}P\left(q,t\right)\right) \end{array}$$ (3)

Here, *R*_*q*′ *q*_ and *R*_*qq*′_ are transition rates of **R** that represent the impact of different mitochondrial processes. The rate *R*_*q*′ *q*_ represents the probability *q* gains by transitions from states *q*′ to *q*, while rate *R*_*qq*′_ depicts the loss of probability from *q* to other states *q′*.

Neglecting possible coupling terms between arbitrary mitochondrial processes *i* and *j*, $\frac{\partial P(q,t)}{\partial t}$ and its corresponding rates *R*_*ij*_ of **R** can be split up into a sum of several terms, each depicting the change in probabilities of *q* by a single mitochondrial process:

$$\begin{array}{c} \frac{\partial }{\partial t}P\left(q,t\right)=\frac{\partial }{\partial t}P_{\text{FF}}\left(q,t\right)+\frac{\partial }{\partial t}P_{\text{MB}}\left(q,t\right)+\frac{\partial }{\partial t}P_{\text{REP}}\left(q,t\right)+\frac{\partial }{\partial t}P_{\text{EC}}\left(q,t\right)+\frac{\partial }{\partial t}P_{\text{ED}}\left(q,t\right) \end{array}$$ (4)

**Fig 1:**
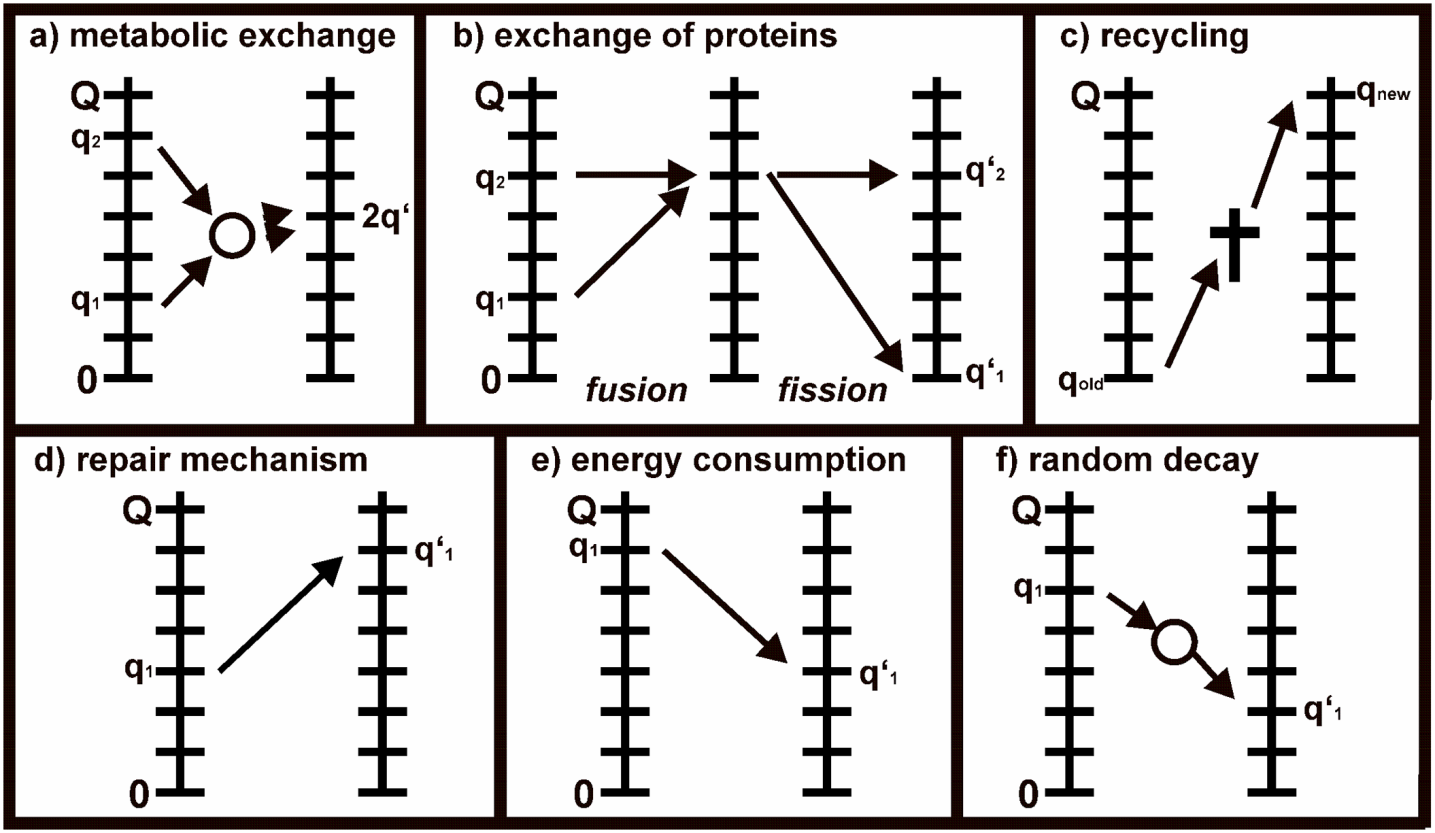
Quality changing processes of the mitochondrial quality model. a) Metabolic fission and fusion process leads to two mitochondria of the same quality. b) Fusion of inner matrix components raises the lower mitochondrium to the quality level of the partner. Fission leads to an inactive mitochondrium while the partner maintains its quality. c) Mitophagy removes inactive mitochondria, biogenesis generates mitochondria of the highest quality. d) Mitochondrial repair renews the quality of mitochondria. e) Energy consumption lowers the quality of highly active mitochondria. f) External damage randomly decreases quality of mitochondria.

The single terms represent mitochondrial quality changing processes which were observed and described in experimental publications in recent years: mitophagy and biogenesis $\frac{\partial }{\partial t}P_{\text{MB}}\left(q,t\right)$, fission and fusion $\frac{\partial }{\partial t}P_{\text{FF}}\left(q,t\right)$, mitochondrial repair $\frac{\partial }{\partial t}P_{\text{REP}}\left(q,t\right)$, energy consumption $\frac{\partial }{\partial t}P_{\text{EC}}\left(q,t\right)$ and external mitochondrial damage $\frac{\partial }{\partial t}P_{\text{ED}}\left(q,t\right)$ (Fig 1).

During aging the number of enzymes, proteins and metabolites alters. As the impact of biological processes depends on the number of corresponding particles or rather molecules involved, the time-dependence of a process *i* can be described by homogeneous differential equation of first order. In biology this approach is chosen, e.g. to derive the effect of a drug over time [25]. The differential equation reads:

$$\begin{array}{c} A_{i}=\pm \frac{dN_{i}}{dt}, \end{array}$$ (5)

where *A*_*i*_ = *λN*_*i*_ denotes the activity of the process and *N*_*i*_ the number of particles involved in the process. This decay law or growth law, depending on the sign, can be solved with an exponential function:

$$\begin{array}{c} \rho_{i}\left(t\right)=\rho_{0,i}\cdot \text{exp}\left(\pm \frac{t}{\tau_{i}}\right), \end{array}$$ (6)

with *ρ*_*i*_(*t*) = *c*⋅*N*_*i*_(*t*), *ρ*_0, *i*_ = *c*⋅*N*_0, *i*_, *τ*_*i*_ = 1/*λ* and *c* being a constant for normalization. This ansatz leads to an decoupling of the mathematical definition *f*_*i*_ of a process and its time-dependent probability *ρ*_*i*_(*t*):

$$\begin{array}{c} \frac{\partial }{\partial t}P_{i}\left(q,t\right)=\rho_{i}\left(t\right)\cdot f\left(P_{i}\left(q,t\right),q,q^{\prime },...\right). \end{array}$$ (7)

Hence, *ρ*_0, *i*_ are independent factors that balance the processes relatively to each other, while *τ*_*i*_ determines the slope of their time-evolutions. The decoupling is true for the processes of mitochondrial fission and fusion, mitophagy and biogenesis and external oxidative stress (see corresponding sections). For the mitochondrial repair mechanism and the process of energy consumption it is coupled to the mathematical definition of the process *i* itself:

$$\begin{array}{c} \frac{\partial }{\partial t}P_{i}\left(q,t\right)=f\left(\rho_{i}\left(t\right),P_{i}\left(q,t\right),q,q^{\prime },...\right). \end{array}$$ (8)

In the following the parameters *ρ*_0, *i*_ and *τ*_*i*_ represent the starting probability and the lifetime of the process *i*, respectively. The sign of *τ*_*i*_ depends on either the increase or the decrease of *i* with the aging of cells.

### Fission and Fusion

The dynamics in a network strongly depend on the mobility of its components. Mitochondria are very motile in the cytoplasm of cells. They are not only transported to different locations [26] but also fuse and divide among each other. [15, 27] These fusion and fission processes exhibit two different patterns. On the one hand there are very quick connections between mitochondria in which only the outer mitochondrial membrane is connected. These so called “kiss and run” patterns are associated with the exchange of rare metabolites among mitochondria. On the other hand more time consuming states of fusion of mitochondria have been found to not only include the outer membrane but also the inner membrane and mitochondrial matrix components. [20] In these fusion states mitochondria are considered to share and compensate for defect protein complexes or DNA sequences which are crucial for the production of ATP. [28] Subsequent fission events lead to one mitochondrium with a still polarized MMP and one mitochondrium with a depolarized MMP. [21]

Considering the two different types of connections among mitochondria we decided to separate the biophysical definition for fission and fusion processes in the model into two parts: metabolic fission and fusion $\frac{\partial }{\partial t}P_{{\text{FF}}_{m}}\left(q,t\right)$ and a proteinaceous fission and fusion $\frac{\partial }{\partial t}P_{{\text{FF}}_{p}}\left(q,t\right)$, leading to

$$\begin{array}{c} \frac{\partial }{\partial t}P_{\text{FF}}\left(q,t\right)=\frac{\partial }{\partial t}P_{{\text{FF}}_{m}}\left(q,t\right)+\gamma \cdot \frac{\partial }{\partial t}P_{{\text{FF}}_{p}}\left(q,t\right), \end{array}$$ (9)

where *γ* denotes a constant to balance both kinds of fission and fusion relatively to each other.

#### Metabolic fission and fusion

To biophysically define the metabolic fission and fusion process (Fig 1a) within our model we make the following assumptions according to literature or biophysical considerations.

1. When two mitochondria with different qualities exchange metabolites no quality can be gained or lost in total: The amount of metabolites remains the same. Thus, for a metabolic fission and fusion event, we suggest a conservation law: $q_{1}+q_{2}\to q_{1}^{\prime }+q_{2}^{\prime }$, with *q*_*i*_ being the incoming qualities and $q_{i}^{\prime }$ the outcoming qualities.
2. For entropic reasons the mixing of metabolites in mitochondria leads to two mitochondria of the same quality: $q_{1}^{\prime }=q_{2}^{\prime }$
3. The larger the discrepancy between the qualities of two mitochondria the more valuable is the exchange of metabolites. Hence, the probability of the process raises with Δ*q* = ∥*q*_1_−*q*_2_∥. Inactive Mitochondria (*q* = 0) are not involved in any fission and fusion exchange of metabolites.

The fission and fusion process of metabolites in the model reads then as follows:

$$\begin{array}{c} \begin{array}{cc} \frac{\partial }{\partial t}P_{{\text{FF}}_{m}}\left(q,t\right)=\rho_{{\text{FF}}_{m}}\left(t\right)\cdot & \sum_{q_{1},q_{2}>0}^{Q}P\left(q_{1},t\right)P\left(q_{2},t\right)R_{{\text{FF}}_{m}}(∥q_{1}-q_{2}∥,t)\;\cdot \\ & \delta_{\left(q_{1}+q_{2}\right),\left(q_{1}^{\prime }+q_{2}^{\prime }\right)}\delta_{q_{1}^{\prime },q_{2}^{\prime }}\left(\delta_{q,q_{1}^{\prime }}+\delta_{q,q_{2}^{\prime }}-\delta_{q,q_{1}}-\delta_{q,q_{2}}\right) \end{array} \end{array}$$ (10)

Here and in the following equations *δ*_*i, j*_ refers to Kronecker’s delta. The two conditions outside the brackets take into account assumption 1 and 2. The positive and negative terms inside the brackets represent the gain and loss of probability of quality state *q*. Assumption 3 is represented by *R*_FF_m__(‖*q*_1_−*q*_2_‖, *t*). This rate is modeled by Hill’s equation, which describes the biochemical process of the binding of a ligand to a macromolecule [29]:

$$\begin{array}{c} R_{\text{F}{\text{F}}_{\text{m}}}(∥q_{1}-q_{2}∥,t)=\frac{∥q_{1}-q_{2}{∥}^{\text{F}{\text{F}}_{1}}}{\text{F}{\text{F}}_{2}{}^{\text{F}{\text{F}}_{1}}+∥q_{1}-q_{2}{∥}^{\text{F}{\text{F}}_{1}}} \end{array}$$ (11)

FF_1_ and FF_2_ are the hill coefficients. They are free parameters that cannot be determined by literature. Later on, we assign them the same values as Figge et al. in their model. The factor *ρ*_FFm_(*t*) represents the time-dependent probability of the process. As the proper balance of fission and fusion is disturbed during the aging of cells [30], the probability of the process decreases with time:

$$\begin{array}{c} \rho_{{\text{FF}}_{m}}\left(t\right)=\rho_{0,{\text{FF}}_{m}}\cdot \text{exp}\left(-\frac{t}{\tau_{{\text{FF}}_{m}}}\right) \end{array}$$ (12)

#### Fission and fusion of proteins

Concerning the fission and fusion of mitochondrial inner matrix components (Fig 1b) we assume according to literature and biophysical considerations the following:

1. As fusion events involving inner matrix components last longer than the exchange of metabolites fission and fusion are seperated into two independent terms
2. When two mitochondria with different qualities share protein complexes after a fusion event both mitochondria have the same capacities to produce energy via oxidative phosphorylation. Hence, the mitochondrium with the lower quality gains the level of quality of the other mitochondrium: *q*_1_+*q*_2_ → 2⋅*q*_1_. Thus, sharing of matrix components is a quality gaining process.
3. The larger the discrepancy between the qualities of two mitochondria the more valuable is the compensation for defect proteins. Hence, similar to the metabolic exchange the probability of a fusion process raises with Δ*q* = ∥*q*_1_−*q*_2_∥. Again, inactive mitochondria (*q* = 0) are not involved in any fusion event.
4. As experimentally observed, after a fission event one mitochondrium keeps the polarization of its MMP while the MMP of the other mitochondrium gets depolarized. [21] In the model, the quality of one mitochondrium remains stable, while the other mitochondrium looses its quality in total: 2⋅*q*_1_ → *q*_1_+0_2_
5. The probability of a fission process increases with lower quality states.

The equation reads as:

$$\begin{array}{c} \begin{array}{cc} \frac{\partial }{\partial t}P_{{\text{FF}}_{p}}\left(q,t\right) & =\rho_{{\text{Fu}}_{p}}\left(t\right)\cdot \sum_{q_{1},q_{2}>0}^{Q}P\left(q_{1},t\right)P\left(q_{2},t\right)R_{{\text{Fu}}_{p}}(∥q_{1}-q_{2}∥,t)\;\cdot \delta_{q_{1},q_{1}^{\prime }}\delta_{q_{1}^{\prime },q_{2}^{\prime }}\left(\delta_{q,q_{2}^{\prime }}-\delta_{q,q_{2}}\right)\\ & +\rho_{{\text{Fi}}_{p}}\left(t\right)\cdot \sum_{q_{1},q_{2}>0}^{Q}P\left(q_{1},t\right)P\left(q_{2},t\right)R_{{\text{Fi}}_{p}}(∥Q-q_{2}∥,t)\;\cdot \delta_{q_{1},q_{2}}\delta_{q_{1},q_{1}^{\prime }}\delta_{q_{2}^{\prime },0}\left(\delta_{q,0}-\delta_{q,q_{2}}\right) \end{array} \end{array}$$ (13)

The first term represents the fusion of inner matrix components and the second term the correspondend fission process. The Kronecker deltas before the brackets refer to the conditions 2 and 3 for fusion and 4 and 5 for fission, respectively. The rates for fusion *R*_Fu_p__(‖*q*_1_−*q*_2_‖, *t*) and fission *R*_Fi_p__(‖*Q*−*q*_1_‖, *t*) are:

$$R_{\text{F}{\text{u}}_{\text{p}}}(∥q_{1}-q_{2}∥,t)=\frac{∥q_{1}-q_{2}{∥}^{\text{F}{\text{F}}_{1}}}{\text{F}{\text{F}}_{2}{}^{\text{F}{\text{F}}_{1}}+∥q_{1}-q_{2}{∥}^{\text{F}{\text{F}}_{1}}}$$ (14)

and

$$R_{\text{F}{\text{i}}_{\text{p}}}(∥q_{1}-q_{2}∥,t)=\frac{∥Q-q_{1}{∥}^{\text{F}{\text{F}}_{1}}}{\text{F}{\text{F}}_{2}{}^{\text{F}{\text{F}}_{1}}+∥Q-q_{1}{∥}^{\text{F}{\text{F}}_{1}}}$$ (15)

Similarly to metabolic fission and fusion, the frequency of networking processes *ρ*_Fu_p__(*t*) decreases and the number of fission events *ρ*_Fi_p__(*t*) increases with the aging of the cell, representing perturbations of networking among mitochondria:

$$\begin{array}{c} \rho_{{\text{Fu}}_{p}}\left(t\right)=\rho_{0,{\text{FF}}_{p}}\cdot \text{exp}\left(-\frac{t}{\tau_{{\text{FF}}_{p}}}\right) \end{array}$$ (16)

$$\begin{array}{c} \rho_{{\text{Fi}}_{p}}\left(t\right)=\rho_{0,{\text{FF}}_{p}}\cdot \text{exp}\left(+\frac{t}{\tau_{{\text{FF}}_{p}}}\right) \end{array}$$ (17)

### Mitophagy and Biogenesis

Mitophagy and mitochondrial biogenesis represent the mitochondrial recycling mechanism of the cell (Fig 1c). Mitochondria that are heavily damaged in terms of their mtDNA or inner and outer mitochondrial membrane components become inactive and establish a depolarized MMP, before they are removed by mitophagy. [31] The removed mitochondrial mass is substituted by new mitochondrial material generated by mitochondrial biogenesis. [32]

Biophysically we define the following rules for mitophagy and biogenesis:

1. To keep the total probability mass at a value of 1 it is required that mitophagy and mitochondrial biogenesis are coupled processes. The equal amount of probability that is subtracted by mitophagy is added by mitochondrial biogenesis: $-\sum_{q=0}^{Q}\frac{\partial }{\partial t}P_{\text{Mitophagy}}\left(q,t\right)=\sum_{q=0}^{Q}\frac{\partial }{\partial t}P_{\text{Biogenesis}}\left(q,t\right)$
2. As mitophagy removes heavily damaged mitochondria with a depolarized MMP from the system we introduce a threshold: Only the probability of the inactive state (*q* = 0) is reduced by mitophagy.
3. Mitochondrial biogenesis generates only mitochondria with the highest possible quality *Q*.

Following these assumptions the probabilistic change by mitophagy and mitochondrial biogenesis is modeled as:

$$\begin{array}{c} \frac{\partial }{\partial t}P_{\text{mb}}\left(q,t\right)=\rho_{\text{mb}}\left(t\right)\cdot \left(-\delta_{q,0}P\left(q,t\right)+\delta_{q,Q}P\left(0,t\right)\right) \end{array}$$ (18)

According to literature mitophagy increases during the aging of cells. [33] Correspondingly, the probability of the processes *ρ*_mb_(*t*) raises with time:

$$\begin{array}{c} \rho_{\text{mb}}\left(t\right)=\rho_{0,\text{mb}}\cdot \text{exp}\left(\frac{t}{\tau_{\text{mb}}}\right) \end{array}$$ (19)

### Repair

For a long time it was not clear whether mitochondria exhibit any strategies to fix damages within their own DNA. Although mitochondria possess a reduced ability to fix DNA damage compared to the nucleus [22], they still have mechanisms to repair defects of the mtDNA, many of them being similar to repair procedures of nuclear DNA. [14] These mechanisms help mitochondria to regain the high production grades of enzymes involved in the Krebs cycle and the integrity of respiration proteins. Due to this background we add a repair process to the model (Fig 1d). The repair algorithm increases the state of quality of mitochondria, thus representing an improvement of the quality of the mtDNA and the production of mitochondrial protein complexes. Despite the evidence that mitochondria utilize repair mechanisms, so far little is known about the selection criteria and the frequency of repair. Therefore, we have to make the following assumption according to biophysical considerations for modeling a repair process:

1. Repair is a stochastic process: Every missing quality is repaired with the same probability. Pursuing this idea with a binomial approach leads to a higher probability of an increase of quality of low quality states.

With a binomial approach, the repair process is biophysically defined as:

∂∂tPrep(q,t)=∑q′=0q′<qQ-q′q-q′ρrep(t)q-q′(1-ρrep(t))Q-q·P(q′,t)-∑q′′>qq′′<qQ-qq′′-qρrep(t)q′′-q(1-ρrep(t))Q-q′′·P(q,t) (20)

The positive term on the right side of the equation refers to all mitochondria with low qualities *q*′ which are repaired to quality state *q*. The negative term takes into account the repair of *q* to higher qualities *q*′′. As the quality of repair can not be conserved during the aging of cells, the probability of repairing a single quality *ρ*_rep_(*t*) decreases with time.

$$\begin{array}{c} \rho_{\text{rep}}\left(t\right)=\rho_{0,\text{rep}}\cdot \text{exp}\left(-\frac{t}{\tau_{\text{rep}}}\right) \end{array}$$ (21)

### Energy consumption

Mitochondria produce ATP via oxidative phosphorylation including the respiration chain. The protein complexes of the respiration chain are located at the inner mitochondrial membrane and are responsible for releasing the energy of NADH molecules in order to establish the proton motive force to transform ADP to ATP. During this process reactive oxygen species (ROS) are generated which harm intramitochondrial structures including the mtDNA. As highly active mitochondria run through the process of oxidative phosphorylation more frequently they suffer more from oxidative stress generated by the electron transport chain than less active mitochondria. [34] We translate this fact to an activity dependent quality decay. Higher mitochondrial quality states which represent more active mitochondria possess a higher probability of loosing quality due to self-generated oxidative stress. With the premise that every mitochondrial quality is lost with the same probability, the probability of quality decay during energy consumption can be modeled with a binomial distribution similar to the repair mechanism (Fig 1e). The quality decay term for energy consumption reads:

∂∂tPec(q,t)=∑q′>qQq′q′-qρec(t)q′-q(1-ρec(t))q·P(q′,t)-∑q′′=0q′′<qqq-q′′ρec(t)q-q′′(1-ρec(t))q′′·P(q,t) (22)

The positive part of the equation refers to a gain of probability of quality *q* by the decrease of the probabilities of higher quality states *q*′ due to internal oxidative stress. Equivalently in the second term quality state *q* looses probability to lower quality states *q*′′. As quality decay by internal ROS damage increases with age due to an increasing amount of mutations in the mtDNA [34], the probability of loosing a specific quality *ρ*_ec_(*t*) increases with time:

$$\begin{array}{c} \rho_{\text{ec}}\left(t\right)=\rho_{0,\text{ec}}\cdot \text{exp}\left(\frac{t}{\tau_{\text{ec}}}\right) \end{array}$$ (23)

### External Damage

Mitochondria do not only damage themselves by oxidative stress, but their mtDNA is also damaged by externally produced ROS from endoplasmatic reticulum, peroxisomes and other organelles in the cell. [35] Moreover, the quality of protein complexes and enzymes involved in the Krebs cycle and the respiration chain is decreased by other externalities leading to the loss of mitochondrial functionalities. These processes affect mitochondria basically in a random manner which is not dependent on the state of metabolic activity of the involved mitochondria. Therefore, we introduce this kind of damage as a quality decreasing process which randomly decreases the quality states of mitochondria to randomly lower levels (Fig 1f).

$$\begin{array}{c} \frac{\partial }{\partial t}P_{\text{ed}}\left(q,t\right)=\rho_{\text{ed}}\left(t\right)\cdot \sum_{q^{\prime }=q+1}^{Q}P\left(q^{\prime },t\right)R_{\text{ed}}\left(q^{\prime }\to q,t\right)-P\left(q,t\right)\sum_{q^{\prime \prime }=0}^{q-1}R_{\text{ed}}\left(q\to q^{\prime \prime },t\right) \end{array}$$ (24)

Here, *R*_ed_ (*q*′ → *q*, *t*) represents the gain term whereas *R*_ed_(*q* → *q*′′, *t*) represents the loss term of the quality state *q*. In order to assure the randomness of the process the algorithm picks random pairs of quality states *q* and *q*′ (and *q*′′, respectively) which exchange a distinct fraction of their probability mass:

$$\begin{array}{c} P\left(q^{\prime },t\right)=P\left(q^{\prime },t\right)\left(1-f_{\text{rd}}\right) \end{array}$$ (25)

and

$$\begin{array}{c} P\left(q,t\right)=P\left(q,t\right)+P\left(q^{\prime },t\right)f_{\text{rd}} \end{array}$$ (26)

with *q*′ > *q* and *f*_rd_ being the lost fraction of *q*′ and *ρ*_ed_(*t*) being the probability of the process. As the production of oxidative stress by other organelles and the impact of external pathogens increase during the aging of the cell, we increase the external damage with time:

$$\begin{array}{c} \rho_{\text{ed}}\left(t\right)=\rho_{0,\text{ed}}\cdot \text{exp}\left(\frac{t}{\tau_{\text{ed}}}\right) \end{array}$$ (27)

## Results

In the following section we present simulations based on the new model on mitochondrial quality states. The differential equation Eq (4) for *P*(*q*, *t*) is numerically solved using Euler’s method for minimal time steps of Δ*t* = 1 tu (tu = time units). Every simulation runs over 100000 tu. At each time step the integrity of the simulation is evaluated by calculating the probability conservation norm. *N*_prob_ has to establish a stable value of 1 to at least the twelfth decimal.

In the original model by Figge et al. the time-dependent change of 11 quality states *q*_*i*_ ∈ {0, 10} with *Q* = 10 was investigated. We choose the same number of states for our simulations. The qualitative results are robust for quality state numbers between 5 (*Q* = 4) and 15 (*Q* = 14). A higher number of states impairs the balance of mitochondrial processes.

The qualitative results of the simulations do not depend on the distribution of probabilities which is fed to the algorithm at the beginning of the simulation. The simulations presented here start with a uniform distribution assigning the same probability to each quality state ($q_{i}=0,\bar{0909}$).

For every point in time *t* we investigate three parameters:

1. average quality: $\bar{q}\left(t\right)=\frac{\sum_{q=0}^{Q}q\cdot P\left(q,t\right)}{Q+1}$
2. deviation of quality: $\sigma_{q}\left(t\right)=\sum_{q=0}^{Q}∥q-\bar{q}\left(t\right)∥\cdot P\left(q,t\right)$
3. fraction of inactive states: *P*(0, *t*)

The model exhibits free parameters which cannot be taken from literature but which have to be estimated. The starting probabilities represent the intrinsic frequency of a specific process, the lifetimes the degree of change of the process during the aging of the cell. These parameters are suitable to descriptively evaluate the consequence of relative changes of mitochondrial processes. In Table 1 we present the values for free parameters in the following simulations. As mitochondrial fission and fusion is a very dynamic process it occurs more frequently than the other processes such as the mitochondrial turnover.[31][36][37][38] Thus, its starting probabilities *ρ*_0, FF_m, p__ are set to values five times higher than the starting probabilities of the other processes. This is in line with the parameter setting in the publication of Figge et al. in 2012. Overall, the parameter values that are taken from the original model are: *ρ*_0, FF_m__, *ρ*_0, FF_p__, FF_1_, FF_2_, *f*_rd_. There is evidence in literature whether the modeled mitochondrial processes rise or fall during the aging of cells as cited in the previous section. However, the frequency of these processes relatively to each other can hardly be determined. In the following we assume that all processes but fission and fusion have the same starting probability to avoid overestimation of a single process according to Laplace’s principle of indifference. This approach concerns all values for the lifetimes *τ*_i_ and the starting probabilities *ρ*_0, mb_, *ρ*_0, rep_, *ρ*_0, ec_, *ρ*_0, ed_. Experiments *in vitro* could help to establish distinct parameter sets for different cell types in order to exactly quantify mitochondrial behavior. The normalization of all lifetimes *τ*_0_ by the lifetime *τ*_i_ of a particularly suitable process would integrate time scalability in the model. Nevertheless, even without experimental determination of the parameter values the simulations remain qualitatively stable as long as the values are not increased or decreased drastically relatively to each other. Even a doubling of starting probabilities and lifetimes does not change the qualitative outcomes in this model (see S1 Fig for examples).

**Table 1:** Values of free parameters in simulations.

parameter	value
*γ*	1
*ρ*_0, FF_m__	0.05
*τ*_FF_m__	50000 tu
*ρ*_0, FF_p__	0.05
*τ*_FF_p__	50000 tu
FF_1_	2
FF_2_	3
*ρ*_0, mb_	0.01
*τ*_mb_	50000 tu
*ρ*_0, rep_	0.01
*τ*_rep_	50000 tu
*ρ*_0, ec_	0.01
*τ*_ec_	50000 tu
*ρ*_0, ed_	0.01
*τ*_ed_	50000 tu
*f*_rd_	0.03

The values were estimated relatively to each other based on literature (see text).

### Single processes

**Fig 2:**
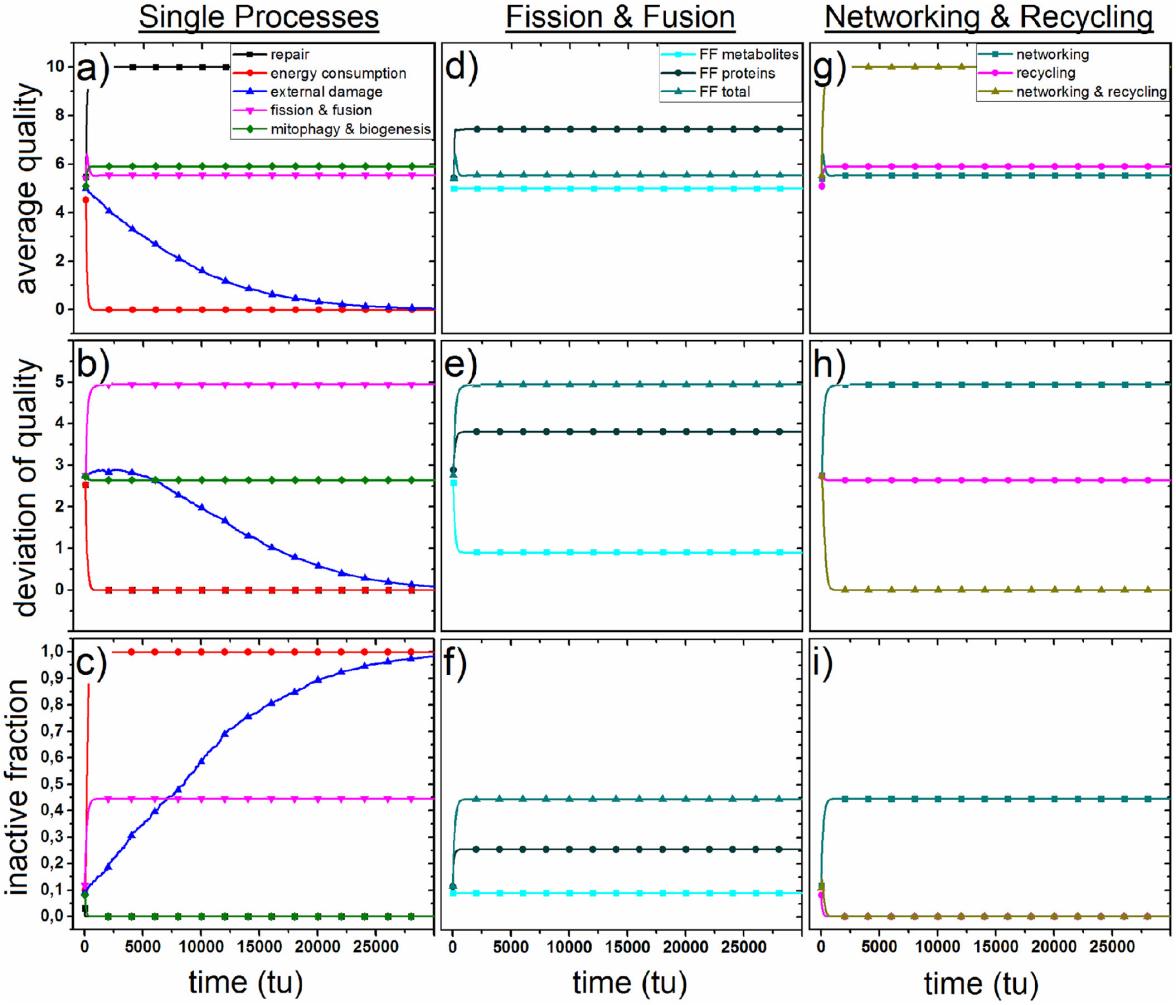
a–c: impact of mitochondrial repair (black), energy consumption (red), external damage (blue), fission and fusion (turquoise) and mitophagy and biogenesis (violett) on the average quality, the deviation of quality and the fraction of inactive states of mitochondria. d–f: impact of metabolic fission and fusion (light turquoise) and proteinaceous fission and fusion (dark turquoise) on average quality, deviation of quality and the inactive fraction compared to the fission and fusion process in total (turquoise). g–h: influence of interplay of networking and recycling (yellow) compared to fission and fusion (turquoise) and mitophagy and biogenesis (violett) on average quality, deviation of quality and inactive fraction.

In Fig 2a and 2c the exclusive impacts of the single processes of Eqs 9, 18, 20, 22 and 24 on average quality $\bar{q}\left(t\right)$, the deviation of quality *σ*_*q*_(*t*) and the fraction of inactive states *P*(0, *t*) are depicted. In terms of $\bar{q}\left(t\right)$ mitochondrial repair is the only quality increasing mechanism while external oxidative stress and energy consumption lead to a decrease of the average quality. Fission and fusion and mitophagy and biogenesis approximately conserve the start value of $\bar{q}\left(t\right)$. The fraction of inactive states vanishes in the presence of only either mitochondrial repair or mitophagy and biogenesis and grows to 1 if either energy consumption and external oxidative stress act exclusively on mitochondria. The fission and fusion process leads to a stable fraction of 0.445 of inactive mitochondria. For logical reasons, *σ*_*q*_(*t*) falls to 0 in the presence of repair, energy consumption or external oxidative stress. Fission and Fusion increases the deviation of quality to about 4.940 while mitophagy and biogenesis keep the value at 2.644 after 30000 tu.

### Fission and fusion

In the mitochondrial quality model presented in this paper the process of fission and fusion is separated into a metabolic and a protein part. In Fig 2d–2f the severed influence of both processes on the quality of mitochondria is presented. The metabolic part changes neither the average quality $\bar{q}\left(t\right)$ nor the number of inactive mitochondria *P*(0, *t*) but decreases the deviation of quality *σ*_*q*_(*t*) to about 0.905. Fission and fusion involving mitochondrial proteins increases all three parameters. The average quality $\bar{q}\left(t\right)$ is raised to 7.441, the number of inactive states *P*(0, *t*) to 0.256 and the deviation of quality *σ*_*q*_(*t*) to 3.808 after 30000 tu.

### Interplay of recycling and networking

In the following mitophagy and biogenesis is refered to as ‘recycling’ and to fission and fusion as ‘networking’. Fig 2a depicts that mitochondrial networking on the one hand and mitochondrial recycling on the other hand are quality conserving processes. In Fig 2g–2i the interplay of both processes in absence of other processes is depicted. A coupling of recycling and networking leads to a drastic increase of the average quality $\bar{q}\left(t\right)$ to 10, and a decrease of both, *σ*_*q*_(*t*) and *P*(0, *t*) to 0 after 30000 tu.

### Mitochondria during cell aging

**Fig 3:**
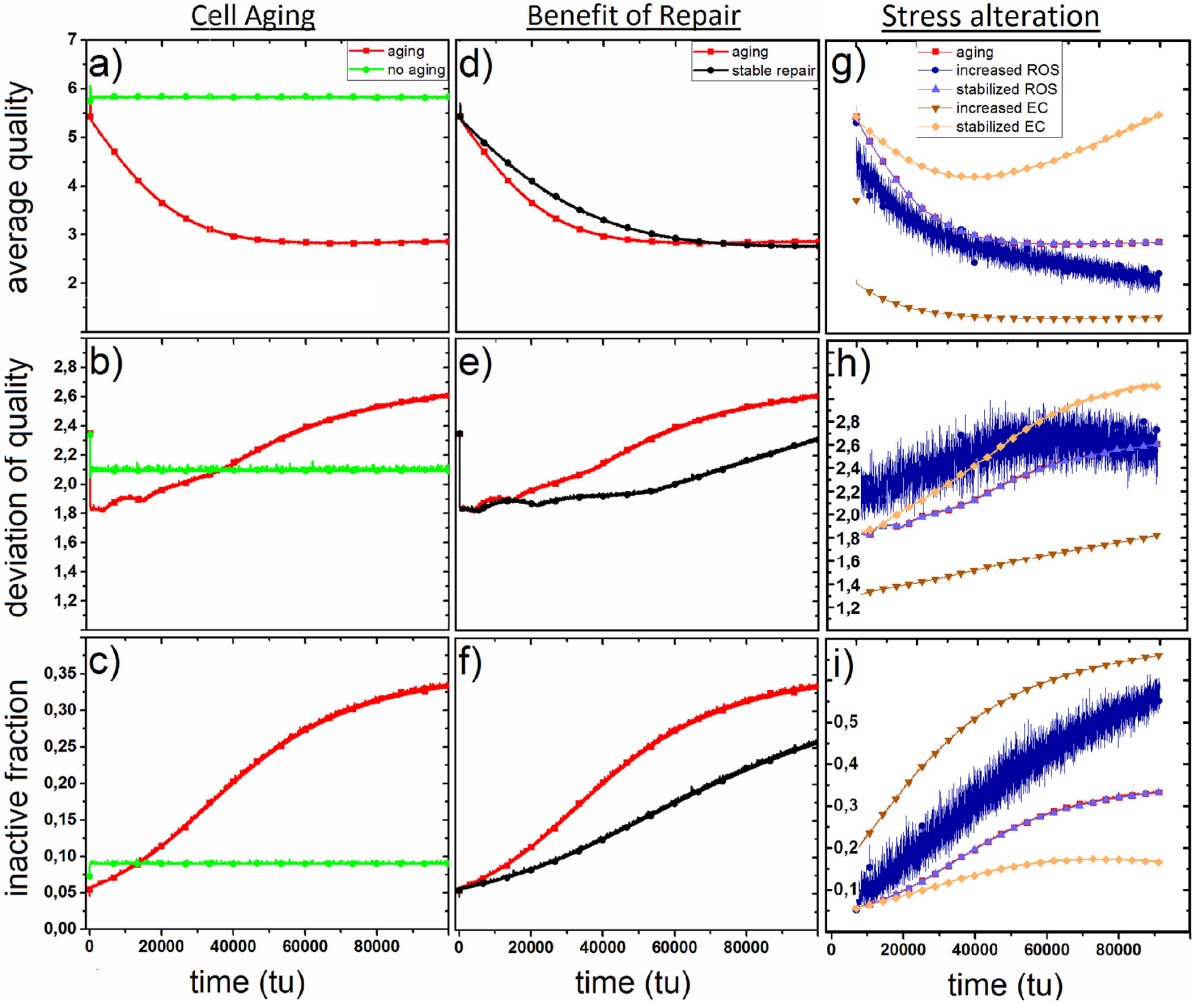
a–c: Comparison of impact of a aging (red) and non-aging (green) of mitochondrial processes on average quality, deviation of quality and fraction of inactive mitochondria with values for free parameters given in Table 1. d–f: Comparison of an aging process with physiologically decreasing repair (red) and an aging process with physiologically stable repair (black) concerning average quality, deviation of quality and fraction of inactive states. g–i: Alteration of parameters in energy consumption (increase of *ρ*_0, ec_ in dark brown, temporal stabilization in light brown) and external damage (increase of *ρ*_0, ed_ in dark blue, temporal stabilization in light blue) concerning average quality, deviation of quality and fraction of inactive states compared to aging (red).

Fig 3a–3c depicts the time-dependence of mitochondrial qualities if all processes act parallel on mitochondria. This simulation represents the evolvement of the mitochondrial network during the aging of cells. For comparison a non-aging cell with stable, time-independenct processes is depicted. To simulate stability in time the lifetimes of all processes were set to *τ*_*i*_ = 500000000. During aging the average quality $\bar{q}\left(t\right)$ falls to a value of about 2.813 at 6900 tu. Then, $\bar{q}\left(t\right)$ slightly increases again to about 2.869 at 100000 tu. The number of inactive states *P*(0, *t*) increases monotonously to fraction of 0.334 at 100000 tu. After a short period of fluctuation the deviation of quality *σ*_*q*_(*t*) starts at about 1450 tu to increase monotonously to 2.611 at 100000 tu. The non-aging process leads to stable values over time for all parameters: $\bar{q}\left(t\right)=5.850$, *σ*_*q*_(*t*) = 2.134 and *P*(0, *t*) = 0, 095.

### Stable repair

We compare the physiologically decreasing repair mechanism with a theoretically stable repair. In order to simulate a stable repair mechanism the lifetime *τ*_0, rep_ is set to 500000000 tu. A comparison of these two simulations (Fig 3d–3f) reveals that only until about 70000 tu a stable repair mechanism is superior to decreasing mitochondrial repair in terms of the average quality $\bar{q}\left(t\right)$. From that point in time on a decreasing repair mechanism leads to a higher average quality than stable mitochondrial repair. The fraction of inactive states *P*(0, *t*) and the deviation of quality *σ*_*q*_(*t*) is lower for stable mitochondrial repair at all points in time.

### Change of ROS production and metabolic activity

In these simulations we investigate the behavior of mitochondrial quality if the conditions of external ROS production and metabolic activity of mitochondria alter. For that purpose, the starting probabilities of the energy consumption is increased to *ρ*_0, ec_ = 0.05, while *ρ*_0, ed_ and *f*_rd_ are set to 0.1 and 0.3, respectively. Furthermore, we simulate a stabilization of both processes over time by setting the lifetimes *τ*_ec_ and *τ*_ed_ to 500000000 tu. Simulations show that an increase of production of oxidative stress as well as a higher metabolic activity (3g-i) leads to a faster loss of quality of mitochondria over time. The increased fluctuation of the dark blue plot is the result of the increased randomization of the production of ROS by externalities. Stabilizing the energy consumption process helps the network to sustain its integrity and even increase it in aged cells. Stabilizing the external damage has no effect on mitochondrial qualities.

### High energy demanding cells

Here, we present different strategies cells with high energy demands could apply to sustain their mitochondrial quality. High energy demanding cells (hec) establish an increased oxidative phosphorylation in order to cope with their high demands for ATP. This mechanism leads to a higher production of reactive oxygen species in mitochondria.

**Fig 4:**
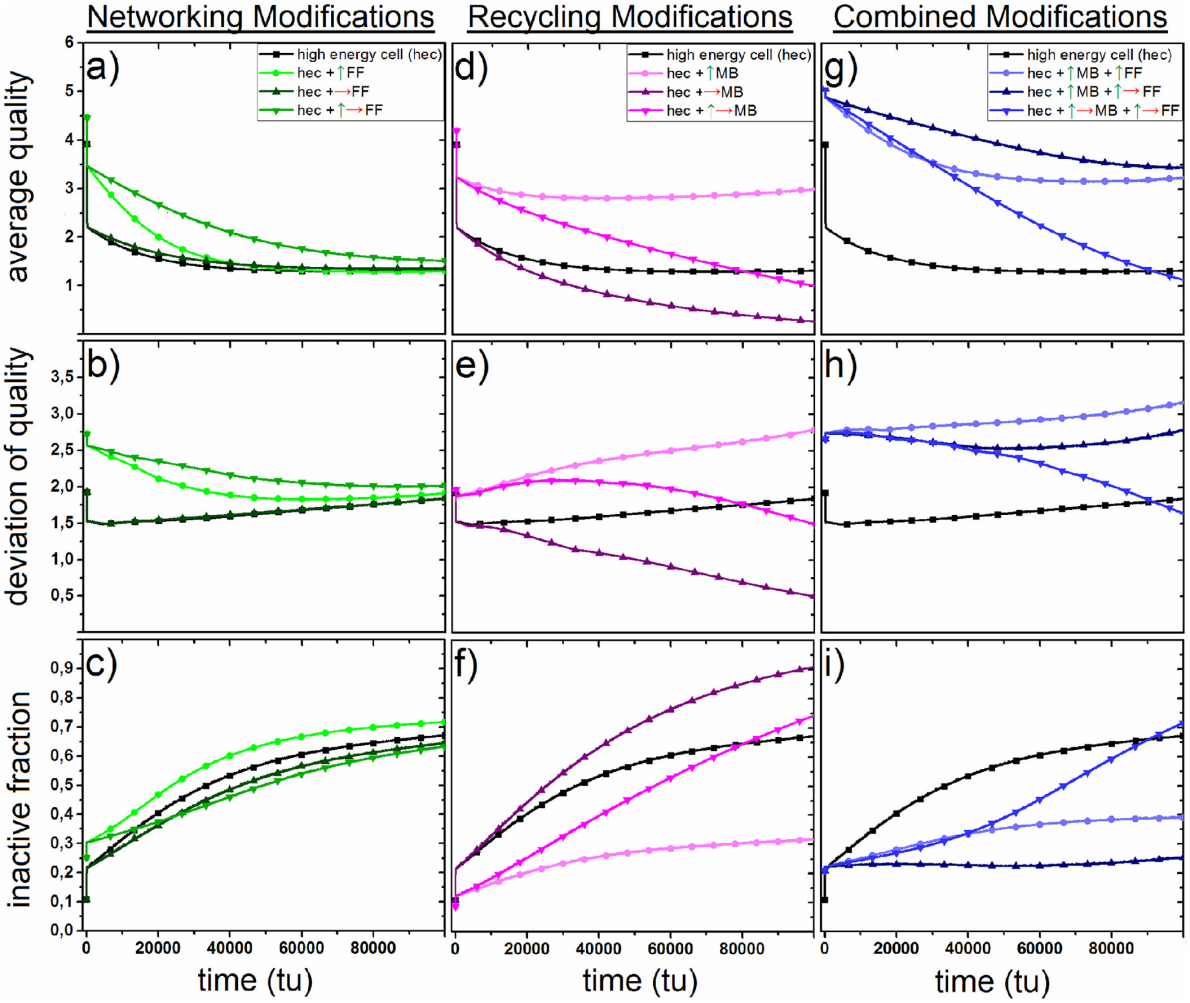
a–c: Development of mitochondrial average quality, deviation of mitochondrial quality and fraction of inactive mitochondria during the aging of a cell with high energy demand (*ρ*_0, ec_ = 0.05, black). The turquoise graphs represent modifications in fission and fusion parameters in high energy demanding cells. (green arrow: *ρ*_0_, FF_m, p_ = 0.5, red arrow: τFF = 500000000). d–f: The violet graphs represent modifications in mitophagy and biogenesis parameters in high energy demanding cells. (green arrow: *ρ*_0_, mb = 0.05, red arrow: τmb = 500000000). g–i: The blue graphs depict simulations with modifications in both, mitochondrial networking and mitochondrial recycling with same parameter alterations as above.

Therefore, we raise the starting probability *ρ*_0, ec_ to 0.05, representing an increased mitochondrial damage induced by internal oxidative stress. The black graphs in Fig 4a–4i indicate the time-dependence of the three quality parameters in high energy demanding cells. The average quality $\bar{q}\left(t\right)$ rapidly decreases with time and asymptotically approaches a value of about 1.650, the deviation of quality *σ*_*q*_(*t*) increases during the aging of the cell to about 1.800 and the inactive fraction *P*(0, *t*) grows to 0.650 after 100000 tu. We simulated several possible alterations of the networking and recycling processes in order to compensate for the loss of quality. The starting probabilities *ρ*_0, mb_ and/or *ρ*_0, FF_m, p__ of were raised by five times and/or the temporal change was prohibited by increasing the lifetimes *τ*_mb_ and/or *τ*_FF_mathrmm, p__ to 500000000 tu. In the plots of Fig 4 these changes are indicated by green arrows (increase of starting probabilities) and a red arrows (increase of lifetimes).

In Fig 4a–4c alterations of the parameters of the networking process in high energy demanding cells are presented. The single increase of the starting probability and the single temporal stabilization of the process leads only to marginal changes in the three quality parameters. The single raise of the starting probability induces only a slight increase of the average quality and a bigger increase of the deviation of quality at the beginning of the simulations. A change of both, *ρ*_0, FF_m, p__ and *τ*_FF_m, p__ leads to bigger increases of $\bar{q}\left(t\right)$ and *σ*_*q*_(*t*) at the beginning of the simulation which decline over time and a slight decrease of the inactive fraction of mitochondria *P*(0, *t*) compared to an unmodified high energy demanding cell.

Fig 4d–4f depicts modifications of the parameters of the recycling process in high energy demanding cells. An increase of the starting probability raises drastically the average quality and the deviation of quality. Additionally, the inactive fraction of mitochondria decreases. These changes are stable in time. A temporal stabilization of the recycling process results in a very low average quality and an increase of *P*(0, *t*) to about 0.9 after 100000 tu. A combination of the alterations in both parameters leads to a increase of $\bar{q}\left(t\right)$ at the beginning of the simulation which then drastically falls to lower values. In the same drastic manner the inactive fraction *P*(0, *t*) climbs to about 0.7 at 100000 tu.

In Fig 4g–4i simulations with alterations of parameters in both processes are presented. Increases of starting probabilities of both networking and recycling lead to an increase of the average quality as well as the deviation of quality. Moreover, the inactive fraction of mitochondria is lower than in a unmodified high energy demanding cell. For simulations with a change of starting probabilities and lifetimes in both processes, networking and recycling, simulations show a high increase in average quality and few inactive mitochondria at the beginning of the simulations. However, the average quality decreases with time while the inactive fraction increase. This decrease of quality is attenuated in simulations with no temporal stabilization of the recycling process but only changes of *ρ*_0, FF_m, p__, *ρ*_0, mb_ and *τ*_FF_m, p__. In these simulations the average quality establishes the highest values and the inactive fraction the lowest values during all points in time compared to all other simulations. The deviation of quality developes a moderate level compared to other modifications.

## Discussion

During the aging of cells mitochondria run through various mitochondrial dynamics. In recent years simulations based on biophysical models of mitochondrial network structures have been performed in order to detect underlying mechanisms behind perfunctory mitochondrial processes. In the new mitochondrial model presented in this publication, five distinct mitochondrial processes were taken into account: A recycling mechanism consisting of mitochondrial biogenesis and mitophagy, mitochondrial networking represented by fission and fusion processes involving proteins and metabolites, internal oxidative stress depending on the degree of the metabolic activity of a mitochondrium as well as stochastic external oxidative stress. Additionally, a repairing process was introduced which represents the regain of mtDNA integrity. The original model of Figge et al. included a mitochondrial process called infectious molecular damage which was designed to take into account the spreading of mtDNA mutations during fission and fusion processes. However, there is no evidence in literature that impairing mtDNA mutations are propagated by mitochondrial networking. Although it was observed that after a fission process one daughter mitochondrium establishes a depolarized MMP, this is not connected to the spreading of intramitochondrial damage but to the induction of mitophagy and the corresponding mitochondrial recycling mechanism. [31]

We investigated three mitochondrial quality parameters: The average quality $\bar{q}\left(t\right)$ represents the well-being of the mitochondrial network in total. The deviation of quality *σ*_*q*_(*t*) gives a degree of how equally the overall quality is distributed among mitochondria. The higher *σ*_*q*_(*t*), the more the qualities in the mitochondrial network are polarized. The third parameter *P*(0, *t*) represents the fraction of inactive mitochondria. Mitochondria in this state are not involved in any networking processes of fission and fusion of neither metabolites nor proteins. These mitochondria lie isolated in the cell and wait for either the repair mechanism to restore their integrity or the mitophagy to remove them. Hence, the fraction of inactive mitochondria represents the degree of fragmentation of the mitochondrial network.

Several free parameters such as lifetimes and starting probabilities of mitochondrial processes have to be chosen in advance of the simulations. The values for these parameters can hardly be determined by literature as many publications handle with different cell types which differentiate regarding the velocity of their internal processes. Furthermore, many of these parameters cannot be measured directly. Thus, the free values of the simulations were estimated relatively to each other. Considering this, the model is fertile for qualitative analysis but is of limited use for exact quantifications.

Additionally, it has to be taken into account that there is no evidence regarding a coupling of mitophagy and biogenesis concerning the total mitochondrial mass. In this model the coupling of both processes is necessary in order to maintain the overall probability at a constant value of 1. Actually, contrary to the increasing mitochondrial autophagy there are indications that aging might degenerate the cell’s ability to perform mitochondrial biogenesis. [30] In order to perform more exact quantifications of the mitochondrial mass in future, one has to suspend the probabilistic approach from the model and substitute it with absolute values.

### Influence of single processes

At first, we investigated the single impact of the individual processes which are defined in the mitochondrial quality model. For most of them their influence on mitochondrial qualities is rather intuitive. The repair mechanism increases the average mitochondrial quality to the maximum value of 10, implying that all mitochondria establish the highest quality state. For logical reasons the fraction of inactive states and the deviation of quality fall correspondingly to 0. Hence, the repair mechanism leads to a mitochondrial network with a maximum of interconnections and mitochondria of high quality without exception. In contrast, internal oxidative stress due to energy consumption by mitochondria and random external oxidative stress generated by other organelles in the cell lead to an average quality of 0. The decrease of the average quality happens to be faster for internalities than for externalities stress implying that mitochondria are more damaged by themselves than by other cell organelles. Intuitively, both processes increase the fraction of inactive states to 1 and decrease the deviation of quality to 0. Hence, both, internal and external oxidative stress lead to a fully fragmented mitochondrial network consisting only of inactive mitochondria.

### Interplay of mitophagy and recycling

Mitophagy and biogenesis are on their own only slightly quality increasing processes in terms of the average quality. After removing all inactive mitochondria from the mitochondrial network mitophagy is not able to remove other mitochondria so that biogenesis can not generate mitochondria with high qualities. In other words: The recycling process stagnates if there are no inactive mitochondria with quality state 0 present in the mitochondrial network. In order to keep the mitochondrial recycling process running mitochondrial fission and fusion generates a significant amount of inactive mitochondria. Investigating networking separately for metabolites and proteins we observe different impacts on mitochondrial quality. Metabolic fission and fusion changes neither the average quality nor the fraction of inactive states but decreases the deviation of quality. This process helps to spread metabolites throughout the cell and increases the corresponding entropy. Fission and fusion of proteins increases moderately the average quality, the deviation of quality and the fraction of inactive states. The interplay of both kinds of fission and fusion prevents the quality increase of proteinaceous networking but only maintains the overall quality level of the starting distribution due to the generation of more inactive mitochondria (0.48 (total) vs 0.28 (proteins) vs 0.09 (metabolites). This effect helps to strongly induce the recycling mechanism of mitophagy and biogenesis without loosing overall quality in the mitochondrial network. Although neither recycling nor networking are on their own quality increasing processes, together they increase the overall mitochondrial quality to the maximum value of 10. Accordingly, the fraction of inactive mitochondria and the deviation of quality fall to 0. This interplay of networking and recycling is proposed in literature. It was observed experimentally that after a fission event there is a high probability that one daughter mitochondrion establishes a depolarized membrane potential accompanied by decreased levels of the fusion protein OPA1 while the other daughter mitochondrion remains metabolically active. As preautophagic mitochondria reduce their membrane potential and OPA1 levels, these results suggest that fission events induce recycling processes.[31]. Our simulations support these findings and reveal a connection between two single processes which are not directly connected within the definitions of the mitochondrial quality model.

### Mitochondrial quality during aging

Over time all mitochondrial processes together lead to a decrease of the average mitochondrial quality and an increase of both, fragmentation and deviation of quality of the mitochondrial network. An age-related fragmentation of the mitochondrial network was observed *in vitro* recently. It was shown that in muscle cells of nematode C. elegans mitochondrial morphologies fragment with increasing age in terms of smaller mitochondrial volumes.[18] Additionally, investigations of cultured skin fibroblasts revealed less elongated mitochondria in cells of old volunteers than of young volunteers. [19] Hence, these experimental results validate the presented model.

The simulations indicate that the fragmentation is connected to an increasing number of in-active mitochondria which can not all be recycled by mitophagy and biogenesis. The increasing number of inactive mitochondria also explains the observed perturbations of fission and fusion cycles during aging in various experiments. The original model by Figge et al. suggests that decreased networking among mitochondria prevents the spread of infectious mitochondrial damage. However, so far there is no experimental evidence that mitochondria can infect each other by fusion processes. Instead, fission and fusion induces the recycling mechanism by generating inactive mitochondria. As the number of inactive mitochondria grow during aging due to internal and external oxidative stress the networking mechanism becomes obsolete regarding this point. Therefore decelerated networking prevents the generation of inactive mitochondria which are not necessary to induce mitochondrial recycling in aged cells due to the excessive supply of inactive mitochondria by damaging processes of internal and external oxidative stress. Interestingly, in aged cells the average quality of mitochondria exhibits asymptotic behavior and remains stable at an average quality of about 3 with even a slight increase to higher qualities. As the fraction of inactive mitochondrial states increases monotonously during aging of cells mitochondrial fragmentation does not necessarily correlate to a decrease of the well-being of the mitochondrial network. Hence, fragmentation might be a quality saving process at some point.

### The benefit of decreasing mitochondrial repair

The slight but unexpected increase of mitochondrial qualities in aged cells can paradoxically be explained with the decrease of mitochondrial repair during aging. A comparison of the aging process with simulations of stable mitochondrial repair reveals that a non-decreasing repair mechanism is only quality saving until a certain point in time. Afterwards the actual aging process is even superior to stable repair. This paradoxical result is explained by a comparison of the fraction of inactive mitochondria in both simulations. The number of inactive mitochondria is constantly lower for the stable repair process even after having a lower average quality than the actual aging simulation. This indicates that the repair mechanism saves mitochondria from becoming inactive so that they cannot be removed by mitophagic processes and recycled by mitochondrial biogenesis. Thus, in an aged cell a stable repair mechanism thwarts mitochondrial recycling compared to a decreased repair mechanism. This context explains the slight increase of the average mitochondrial quality at the end of the aging simulation. The decreased repair mechanism fails in preventing mitochondria from becoming inactive. Thus, there is a higher probability for mitochondrial recycling to replace the inactive mitochondria with high quality mitochondria that increases the overall quality the mitochondrial network.

### Mitochondrial qualities in stressed cells

Environmental circumstances such as high UV radiation or pathogens occasionally require cells to perform high metabolic activities and stimulate the production of reactive oxygen species. These circumstances are simulated by increasing probabilities of external damage and energy consumption. Simulations show that stress leads to degradation of mitochondrial qualities as well as a higher number of isolated mitochondria revealing growing mitochondrial fission states. These results are validated by experimental observations on human lung adenocarcinoma cells. Under the condition of oxidative stress induced by high-fluence low-power laser radiation these cells display a fragmented structure of the mitochondrial network.[39]

Consequently, a stabilization of the production of reactive oxygen species by electron transport chain helps mitochondria to sustain a high quality level and to prevent a high number isolated mitochondria and a fragmented mitochondrial network, respectively. Under this condition we even observe an increase of mitochondrial qualities in aged cells. The reason for this unphysiological behavior might be that in the model so far a coupling of recycling and oxidative stress is not included. It is questionable if the frequency of mitophagy is still increasing in aged cells if oxidative stress is kept at a low level. Therefore, a future expansion of the differential equation in the model should implement a coupling term between recycling and oxidative stress in order to take into account the corresponding feedback.

### Sustaining mitochondrial quality in high energy demanding cells

Many active cell types in human tissue of liver, heart or brain require an elevated supply with ATP in order to maintain their functioning. We simulated those cells with high energy demands by increasing their probability of producing internal oxidative stress. Consequently, mitochondria in energy demanding cells harm their own integrity more than cells with a common energy metabolism. This leads to a low quality within the mitochondrial network accompanied by a high fraction of inactive mitochondria that increases during the aging of the cell. As many cells such as hepatocytes or neurons [40], [41] rely on a constant and high-quality supply with energy, mitochondria in these cells have to develop strategies to handle the loss of quality due to their internal oxidative stress. Two processes which appear to be at least partially controlled by mitochondria to sustain their quality are their networking and their recycling. Hence, we modified the free parameters of these processes to identify mechanisms that establish a healthy quality level in mitochondria of energy demanding cells.

Simulations show that isolated single changes in neither starting probability nor the temporal attenuation of networking lead to a distinct increase in the average quality among mitochondria. A combination of an alteration of both parameters results in a moderate increase of average qualities and a corresponding decrease of inactive mitochondria at the beginning of the simulation. This improvement in qualities falls during the aging of cells. Nevertheless it appears to be a valid strategy for mitochondria in energy demanding cells, to increase their probability for fission and fusion and to a stabilize a high level of networking during the aging of the cell in order to keep a high level of quality within the mitochondrial network at least at the beginning of its lifetime. However, this mechanism is accompanied by an increased deviation of quality among mitochondria. This observation suggests that the mechanism helps to generate high quality states of mitochondria at specific locations of high energy demands within the cell but it does not support maintaining a high quality level within the whole mitochondrial network. While a temporal stabilization of the recycling process leads to a drastic decrease of average qualities, an increase of the starting probability for recycling results in a significant increase of the qualities of mitochondria. The improvement is stable during the aging of the cell.

Adding this increase of the probability for recycling to the combined changes in parameters of networking, mitochondria establish an even higher average quality with less deviation in quality and a low fraction of inactive mitochondria. Compared to this strategy, other modifications on networking and recycling are either not significantly quality increasing or have restrictions in terms of their time dependence. Attenuating the temporal change in both processes, networking and recycling as well as an increase in the starting probabilities of both processes increases qualities of mitochondria the beginning of the lifetime of the cell, but this improvement cannot be maintained during aging. Hence, attenuating the decrease of networking processes in addition to a higher starting probabilities of networking and recycling appears to be the best strategy in order to keep a high, widely spread quality level among mitochondria in energy demanding cells.

### Conclusion

In conclusion, we introduced a novel mitochondrial quality model based on the approach of Figge et al in 2012. In the model we transfer the latest findings of experimental research on mitochondrial processes during the aging of cells into explicitly defined biophysical processes. Additionally, we introduce a universal decay and growth law for each mitochondrial process which describes its time-dependence during the aging of cells. Simulations are in coherence with experimental investigations of the mitochondrial network and support current hypotheses about the interplay of distinct mitochondrial processes, revealing new mechanisms that influence mitochondrial qualities during the aging of cells. Our model proposes a fragmentation of the mitochondrial network during aging, suggests a quality increasing coupling of mitochondrial recycling and networking and displays a quality saving mechanism by the decrease of mitochondrial repair functionalities in aged cells. Furthermore, simulations propose that temporal stabilization of networking accompanied by an increase of probabilities in recycling and fission and fusion is a significant quality saving strategy in cells with high energy demands. Overall, the revealed findings give new insights in mitochondrial processes during aging, providing suggestions for further experimental investigations in future.

## Supporting Information

S1 Fig a–c: Interplay of networking and recycling with modified parameters in networking (turquoise, *ρ*_0, FF_m, p__ = 0.1, *τ*_FF_m, p__ = 100000) tu and recycling (violet, *ρ*_0, mb_ = 0.02, *τ*_mb_ = 100000) tu. Apart from slight differences at the beginning of each simulation the parameter modifications do not change the qualitative outcome presented in Fig 2d–2f. d–f: Temporal stabilized repair mechanism (dark grey) with modified parameters (*ρ*_0, rep_ = 0.02) compared with an unmodified aging process. The point in time at which natural aging is superior to a stabilized repair mechanism is delayed in this simulation to about 100000 tu but the qualitative outcome in Fig 3d–3f is confirmed.
(TIF)

## References

[1] Figge MT, Reichert AS, Meyer-Hermann M, Osiewacz HD. Deceleration of fusion-fission cycles improves mitochondrial quality control during aging. PLoS Comput Biol. 2012;8(6):e1002576 10.1371/journal.pcbi.1002576 22761564 PMC3386171

[2] Miquel J, Economos AC, Fleming J, Johnson J J E. Mitochondrial role in cell aging. Exp Gerontol. 1980;15(6):575–91. 10.1016/0531-5565(80)90010-8 7009178

[3] Balaban RS, Nemoto S, Finkel T. Mitochondria, oxidants, and aging. Cell. 2005;120(4):483–95. 10.1016/j.cell.2005.02.001 15734681

[4] Lodish H. Molecular Cell Biology. Freeman, W. H. & Company; 2004.

[5] Leyssens A, Nowicky AV, Patterson L, Crompton M, Duchen MR. The relationship between mitochondrial state, ATP hydrolysis, [Mg2+], and [Ca2+](i) studied in isolated rat cardiomyocytes. Journal of Physiology-London. 1996;496(1):111–128. 10.1113/jphysiol.1996.sp021669 PMC1160828 8910200

[6] Yakes FM, Van Houten B. Mitochondrial DNA damage is more extensive and persists longer than nuclear DNA damage in human cells following oxidative stress. Proc Natl Acad Sci U S A. 1997;94(2):514–9. 10.1073/pnas.94.2.514 9012815 PMC19544

[7] Shokolenko I, Venediktova N, Bochkareva A, Wilson GL, Alexeyev MF. Oxidative stress induces degradation of mitochondrial DNA. Nucleic Acids Res. 2009;37(8):2539–48. 10.1093/nar/gkp100 19264794 PMC2677867

[8] Lin MT, Beal MF. Mitochondrial dysfunction and oxidative stress in neurodegenerative diseases. Nature. 2006;443(7113):787–95. 10.1038/nature05292 17051205

[9] Busch KB, Kowald A, Spelbrink JN. Quality matters: how does mitochondrial network dynamics and quality control impact on mtDNA integrity? Philos Trans R Soc Lond B Biol Sci. 2014;369(1646):20130442 24864312 10.1098/rstb.2013.0442 PMC4032518

[10] Nakada K, Inoue K, Ono T, Isobe K, Ogura A, Goto YI, et al Inter-mitochondrial complementation: Mitochondria-specific system preventing mice from expression of disease phenotypes by mutant mtDNA. Nat Med. 2001;7(8):934–40. 10.1038/90976 11479626

[11] Rolland SG, Motori E, Memar N, Hench J, Frank S, Winklhofer KF, et al Impaired complex IV activity in response to loss of LRPPRC function can be compensated by mitochondrial hyperfusion. Proc Natl Acad Sci U S A. 2013;110(32):E2967–76. 10.1073/pnas.1303872110 23878239 PMC3740885

[12] Kim I, Rodriguez-Enriquez S, Lemasters JJ. Selective degradation of mitochondria by mitophagy. Arch Biochem Biophys. 2007;462(2):245–53. 10.1016/j.abb.2007.03.034 17475204 PMC2756107

[13] Jornayvaz FR, Shulman GI. Regulation of mitochondrial biogenesis. Essays Biochem. 2010;47:69–84. 10.1042/bse0470069 20533901 PMC3883043

[14] Larsen NB, Rasmussen M, Rasmussen LJ. Nuclear and mitochondrial DNA repair: similar pathways? Mitochondrion. 2005;5(2):89–108. 16050976 10.1016/j.mito.2005.02.002

[15] Youle RJ, van der Bliek AM. Mitochondrial fission, fusion, and stress. Science. 2012;337(6098):1062–5. 10.1126/science.1219855 22936770 PMC4762028

[16] Patel PK, Shirihai O, Huang KC. Optimal dynamics for quality control in spatially distributed mitochondrial networks. PLoS Comput Biol. 2013;9(7):e1003108 10.1371/journal.pcbi.1003108 23874166 PMC3708874

[17] Mouli PK, Twig G, Shirihai OS. Frequency and selectivity of mitochondrial fusion are key to its quality maintenance function. Biophys J. 2009;96(9):3509–18. 10.1016/j.bpj.2008.12.3959 19413957 PMC2711405

[18] Regmi SG, Rolland SG, Conradt B. Age-dependent changes in mitochondrial morphology and volume are not predictors of lifespan. Aging (Albany NY). 2014;6(2):118–30. 24642473 10.18632/aging.100639 PMC3969280

[19] Sgarbi G, Matarrese P, Pinti M, Lanzarini C, Ascione B, Gibellini L, et al Mitochondria hyperfusion and elevated autophagic activity are key mechanisms for cellular bioenergetic preservation in centenarians. Aging (Albany NY). 2014;6(4):296–310. 24799450 10.18632/aging.100654 PMC4032796

[20] Liu X, Weaver D, Shirihai O, Hajnoczky G. Mitochondrial ’kiss-and-run’: interplay between mitochondrial motility and fusion-fission dynamics. EMBO J. 2009;28(20):3074–89. 10.1038/emboj.2009.255 19745815 PMC2771091

[21] Twig G, Shirihai OS. The interplay between mitochondrial dynamics and mitophagy. Antioxid Redox Signal. 2011;14(10):1939–51. 10.1089/ars.2010.3779 21128700 PMC3078508

[22] Alexeyev M, Shokolenko I, Wilson G, LeDoux S. The maintenance of mitochondrial DNA integrity—critical analysis and update. Cold Spring Harb Perspect Biol. 2013;5(5):a012641 10.1101/cshperspect.a012641 23637283 PMC3632056

[23] Liu Y, Fiskum G, Schubert D. Generation of reactive oxygen species by the mitochondrial electron transport chain J Neurochem. 2002;80(5):780–7. 11948241 10.1046/j.0022-3042.2002.00744.x

[24] Kampen NGv. Stochastic Processes in Physics and Chemistry. 3rd ed North-Holland Personal Library; 2007.

[25] Winter ME. Basic Clinical Pharmacokinetics. 5th ed Lippincott Williams and Wilkins; 2009.

[26] Schwarz TL. Mitochondrial trafficking in neurons. Cold Spring Harb Perspect Biol. 2013;5(6). 10.1101/cshperspect.a011304 23732472 PMC3660831

[27] van der Bliek AM, Shen Q, Kawajiri S. Mechanisms of mitochondrial fission and fusion. Cold Spring Harb Perspect Biol. 2013;5(6). 10.1101/cshperspect.a011072 23732471 PMC3660830

[28] Chen H, Vermulst M, Wang YE, Chomyn A, Prolla TA, McCaffery JM, et al Mitochondrial fusion is required for mtDNA stability in skeletal muscle and tolerance of mtDNA mutations. Cell. 2010;141(2):280–9. 10.1016/j.cell.2010.02.026 20403324 PMC2876819

[29] Hill AV. The possible effects of the aggregation of the molecules of haemoglobin on its dissociation curves. The Journal of Physiology. 1910;40(iv–vii).

[30] Seo AY, Joseph AM, Dutta D, Hwang JC, Aris JP, Leeuwenburgh C. New insights into the role of mitochondria in aging: mitochondrial dynamics and more. J Cell Sci. 2010;123(Pt 15):2533–42. 10.1242/jcs.070490 20940129 PMC2912461

[31] Twig G, Elorza A, Molina AJ, Mohamed H, Wikstrom JD, Walzer G, et al Fission and selective fusion govern mitochondrial segregation and elimination by autophagy. EMBO J. 2008;27(2):433–46. 10.1038/sj.emboj.7601963 18200046 PMC2234339

[32] Palikaras K, Tavernarakis N. Mitochondrial homeostasis: the interplay between mitophagy and mitochondrial biogenesis. Exp Gerontol. 2014;56:182–8. 10.1016/j.exger.2014.01.021 24486129

[33] Oberley TD, Swanlund JM, Zhang HJ, Kregel KC. Aging results in increased autophagy of mitochondria and protein nitration in rat hepatocytes following heat stress. J Histochem Cytochem. 2008;56(6):615–27. 10.1369/jhc.2008.950873 18379016 PMC2386765

[34] Wei YH, Lee HC. Oxidative stress, mitochondrial DNA mutation, and impairment of antioxidant enzymes in aging. Exp Biol Med (Maywood). 2002;227(9):671–82. 12324649 10.1177/153537020222700901

[35] Brown GC, Borutaite V. There is no evidence that mitochondria are the main source of reactive oxygen species in mammalian cells. Mitochondrion. 2012;12(1):1–4. 10.1016/j.mito.2011.02.001 21303703

[36] Lipsky NG, Pedersen PL. Mitochondrial turnover in animal cells. Half-lives of mitochondria and mitochondrial subfractions of rat liver based on [14C]bicarbonate incorporation. J Biol Chem. 1981;256(16):8652–7. 7263675

[37] Menzies RA, Gold PH. The turnover of mitochondria in a variety of tissues of young adult and aged rats. J Biol Chem. 1971;246(8):2425–9. 5553400

[38] Miwa S, Lawless C, von Zglinicki T. Mitochondrial turnover in liver is fast in vivo and is accelerated by dietary restriction: application of a simple dynamic model. Aging Cell. 2008;7(6):920–3. 10.1111/j.1474-9726.2008.00426.x 18691181 PMC2659384

[39] Wu S, Zhou F, Zhang Z, Xing D. Mitochondrial oxidative stress causes mitochondrial fragmentation via differential modulation of mitochondrial fissoin-fusion proteins. FEBS Journal. 2011;278:941–954. 10.1111/j.1742-4658.2011.08010.x 21232014

[40] Berndt N, Holzhutter HG. The high energy demand of neuronal cells caused by passive leak currents is not a waste of energy. Cell Biochem Biophys. 2013;67(2):527–35. 10.1007/s12013-013-9538-3 23479331

[41] Cervinkova Z, Lotkova H, Krivakova P, Rousar T, Kucera O, Tichy L, et al Evaluation of mitochondrial function in isolated rat hepatocytes and mitochondria during oxidative stress. Altern Lab Anim. 2007;35(3):353–61. 17650955 10.1177/026119290703500303

