# Supplementary figures and images for: Quality Saving Mechanisms of Mitochondria during Aging in a Fully Time-Dependent Computational Biophysical Model
Source: PLoS One. 2016 Jan 15;11(1):e0146973. doi: 10.1371/journal.pone.0146973 (PMC4738421; doi:10.1371/journal.pone.0146973)

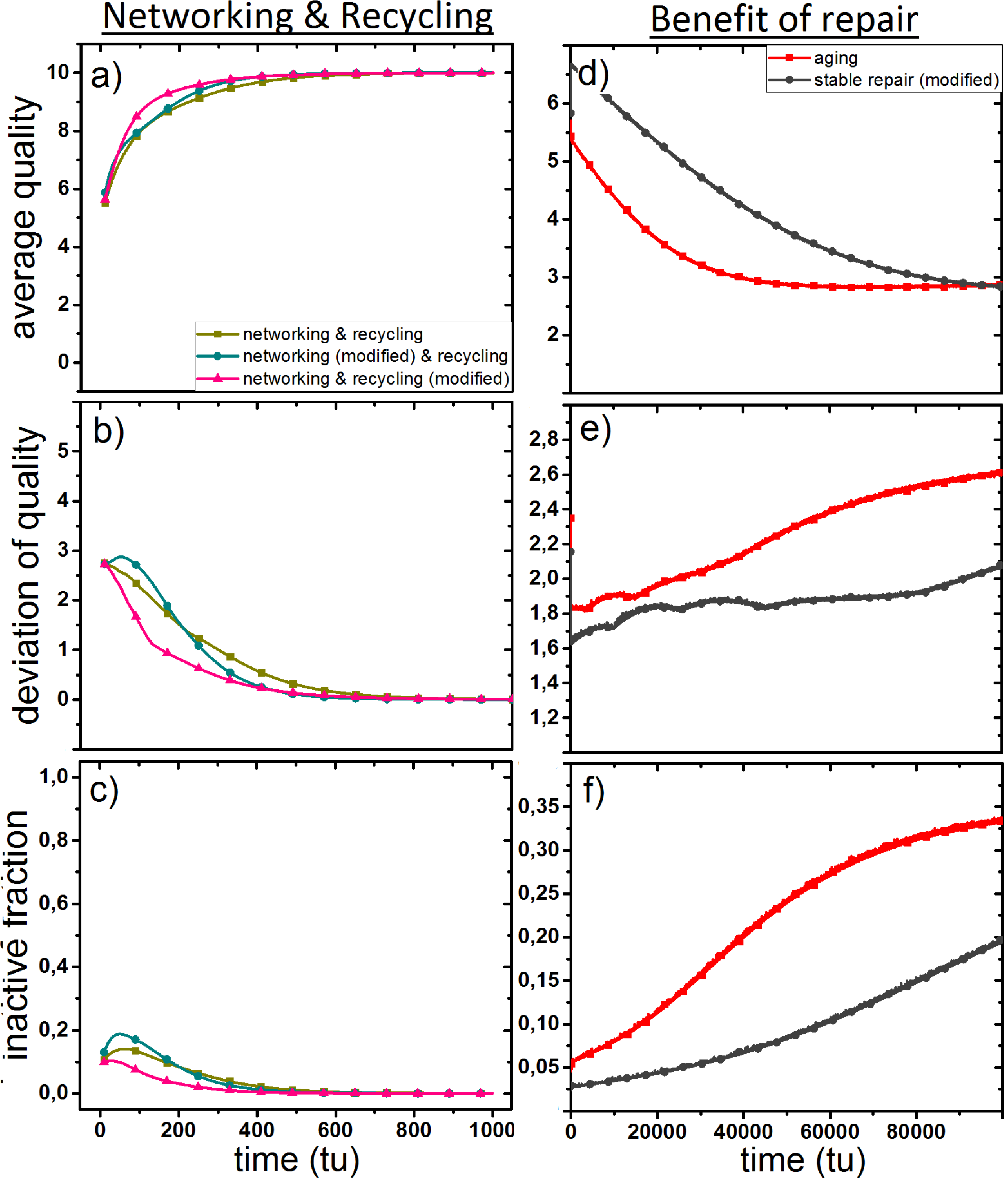

Supplement: S1 Fig — a–c: Interplay of networking and recycling with modified parameters in networking (turquoise, ρ0, FFm, p = 0.1, τFFm, p = 100000) tu and recycling (violet, ρ0, mb = 0.02, τmb = 100000) tu. Apart from slight differences at the beginning of each simulation the parameter modifications do not change the qualitative outcome presented in Fig 2d–2f. d–f: Temporal stabilized repair mechanism (dark grey) with modified parameters (ρ0, rep = 0.02) compared with an unmodified aging process. The point in time at which natural aging is superior to a stabilized repair mechanism is delayed in this simulation to about 100000 tu but the qualitative outcome in Fig 3d–3f is confirmed. (TIF) [file pone.0146973.s001.tif]
